# Problem Behaviors of Adolescents: The Role of Family Socioeconomic Status, Parental Educational Expectations, and Adolescents’ Confidence in the Future

**DOI:** 10.3390/ijerph192315442

**Published:** 2022-11-22

**Authors:** Yanwen Ouyang, Daoqun Ding, Xizheng Xu

**Affiliations:** 1 School of Educational Science, Hunan Normal University, Changsha 410081, China; 2 Department of Management, Hunan Police Academy, Changsha 410138, China

**Keywords:** family socioeconomic status, problem behaviors, educational expectations, confidence in the future, adolescents

## Abstract

The comprehensive theoretical model of problem behaviors proposes that family socioeconomic status is one of the factors affecting adolescent problem behaviors. The purpose of this study is to further explore the mechanism of the influence of family socioeconomic status on adolescent problem behaviors based on the framework of the comprehensive theoretical model of problem behaviors. Further, it is to discover more protective or risk factors affecting adolescent problem behaviors, so as to provide theoretical guidance for the prevention of and intervention in adolescent problem behaviors. This study used data from the 2014–2015 academic year of the China Education Panel Survey (CEPS) conducted by the China survey and data center at the Renmin University of China. A national representative sample of 6888 junior high school students was selected. Among them, 3342 (48.5%) were girls and 3546 (51.5%) were boys with an average age of 14.50 years (SD = 0.68 years). The results revealed that parental educational expectations and adolescents’ confidence in the future played a contributory mediating role in the association between family socioeconomic status and adolescents’ problem behaviors. Both parental educational expectations and adolescents’ confidence in the future are protective factors against adolescents’ problem behaviors and enhancing these factors can decrease the likelihood of adolescents’ engagement in problem behaviors.

## 1. Introduction

Problem behaviors are defined as abnormal behaviors that hamper an individual’s social adaptation [1] and violate social norms or fail to adapt to social life, causing negative influence and even harm to societies and communities [2]. Examples include aggression, school discipline violations, and antisocial behaviors. Problem behaviors affect adolescents’ mental health and hinder their development [3,4]. Problem behaviors that develop in childhood can manifest later in life as adult personality disorders, increasing the likelihood of substance abuse and illegal involvement [5,6,7]. Therefore, it is crucial to explore the antecedent variables of adolescents’ problem behaviors and the associations between those variables.

### 1.1. The Comprehensive Theoretical Model of Problem Behaviors

Influenced by positive psychology, researchers pay attention to the prevention and intervention of problem behaviors, holding to the assertion that problem behaviors usually manifest in the form of a “syndrome”. Thus, problem behaviors should be studied as a whole, not individually [8]; the comprehensive theoretical model of problem behaviors (CTMPB) is one of the crucial theories. In 2003, Jessor et al., established the CTMPB based on two theories: One is the two-factor theory, developed by Rutter, which argues that research on problem behaviors should not only focus on the factors that cause problem behaviors—risk factors—but also focus on the factors that can, directly and indirectly, mitigate problem behaviors—protective factors [9]. The second is the problem-behavior theory, previously developed by Jessor, which argues that there are three psychosocial systems that influence problem behaviors: The personality system, the perceived environment system, and the behavior system [10].

The CTMPB was developed as a risk-protection model, which includes three types of protection (models protection, controls protection, and support protection) and three types of risk (models, opportunity, and vulnerability risks). Models protection includes measures such as parental involvement in communities and peer models for health-enhancing behaviors such as engagement in regular exercise; controls protection includes individual-level measures such as attitudinal intolerance of deviance and social environmental measures such as predictable parental sanctions, and support protection includes contextual measures such as family closeness. Regarding risk, models risk includes measures such as parental smoking and peer models for alcohol use; opportunity risk includes opportunity measures such as the availability of alcohol in the home; and vulnerability risk includes personal vulnerability measures such as feelings of stress and low self-esteem [11]. This explanatory model gives a better account of the problem behavior and goes a step ahead of the two-factor and problem-behavior theories. 

### 1.2. Association between Socioeconomic Status and Problem Behaviors

Family socioeconomic status refers to the rank, hierarchy, and status of a family in a society, which reflects the differences between the current or future resources of a family [12]. The family socioeconomic status of adolescents is often measured by family income, parents’ educational level, and occupation [13]. Jessor pointed out that family socioeconomic status is an antecedent variable affecting adolescents’ problem behaviors. There is much empirical support for Jessor’s view, as well as increasing evidence showing that improvements in family socioeconomic status may induce beneficial effects in children [14,15,16,17,18]. For example, a quasi-experimental study conducted by Costello et al. showed that the increase in parental employment and family income in poor communities was significantly associated with the decrease in adolescents’ problem behaviors [19]. 

However, CMTPB has not explored the mechanism of the influence of family socioeconomic status on adolescents’ problem behaviors. There are two studies on this topic at present, and both are guided by the bioecological theory. One study aimed to explore the psychological mechanisms underlying the relationship between family socioeconomic status (SES) and problem behaviors in Chinese children. According to Bronfenbrenner’s bioecological theory, this study took SES as the exosystem factor and hypothesized that SES influences parental emotional warmth (as a microsystem factor) and consequently, psychological suzhi (as an individual level factor), directly affects problem behaviors (as children’s development factors). Participants were 1128 children (556 females) from two Chinese elementary schools, aged 8–13 years. Children provided self-reports on parental emotional warmth and psychological suzhi, whereas parents reported on SES and problem behaviors. The results of the study showed that parental emotional warmth and individual psychological quality mediate the relationship between family socioeconomic status and problem behaviors [15].

Another study also aimed to explore the effect of family socioeconomic status on adolescent problem behaviors and its underlying mechanism based on the bioecological theory. Similar to the previous study, this study took parental emotional warmth as a microenvironmental system variable, hypothesized that it would directly affect individual belief in a just world as an individual factor, and then affect individual psychological and behavioral development. It used the family socioeconomic status assessment, and the strengths and difficulties questionnaire survey for 1337 adolescents (grades 7–12) in 13 middle schools in the country’s three major regions. The results of this study revealed that parents’ emotional warmth and belief in a just world played a chain-mediating role between family socioeconomic status and problem behaviors [20].

However, although these studies partially explain the influence mechanism of family socioeconomic status on problem behaviors, they are far from sufficient, especially after the emergence of many theoretical models related to family socioeconomic status, such as family investment and family stress theories. Therefore, more studies need to be conducted, and additional variables such as educational resources, parents’ educational concepts, and individual personalities, associated with family socioeconomic status and adolescents’ problem behaviors, should be considered. Therefore, this study aims to further explore the mechanism of the influence of family socioeconomic status on adolescents’ problem behaviors based on the theoretical framework of CTMPB, explore more protective and risk factors that influence adolescents’ problem behaviors, and provide theoretical support for the intervention of adolescents’ problem behaviors.

### 1.3. The Mediating Role of Parental Educational Expectations and Adolescents’ Confidence in the Future

According to the CTMPB, conceptually, protective factors decrease the likelihood of engaging in problem behaviors whereas risk factors increase the likelihood of engaging in problem behaviors. The risk-protection model of the CTMPB includes two kinds of measures of protection and risk, including situational and personality system factors. Situational system factors, known as distal variables, have an indirect impact on problem behaviors. Personality system factors, known as proximal variables, have a more direct impact on problem behaviors [11].

Situational system factors generally originate from multiple social contexts that are salient in the ecology of daily adolescent life: family, peers, school, and neighborhood [11]. Parental educational expectations are a kind of situational system factor from the family and refer to parents’ expectations of what level of education their teenagers should eventually attain [21]. According to the family investment theory, families with high socioeconomic status can provide higher economic, human, and social capital to promote the development of their children, so these parents will have a higher expectation of their children’s education [12]. It has been acknowledged that parental educational expectation affects adolescents’ academic achievement and educational attainment [22]. However, parental educational expectations go far beyond that. There may be a significant correlation between parents’ educational expectations and adolescent problem behaviors. Jessor et al. proposed that self-educational expectations are one of the psychosocial factors associated with adolescents’ problem behaviors [11]. Although there is a great general difference between the self-educational expectation of adolescents and parental educational expectation [23], a large number of previous studies found that there is an intergenerational relationship between the educational level of parents and the educational level of adolescents, and parental educational expectation can significantly predict adolescents’ self-educational expectation [24,25,26]. However, it should be further investigated whether parental educational expectations are protective factors and mediate the relationship between family socioeconomic status and adolescents’ problem behaviors.

The variables constituting the personality system are at the level of social cognition and include values, expectations, beliefs, attitudes, and orientations of self and others [11]. However, not all personality system factors affect problem behaviors. Only the personality variables that have a control effect on problem behaviors may become protective or risk factors. Previous studies have confirmed that self-esteem, as a positive psychological quality, has an important impact on adolescents’ problem behaviors [27], and high self-esteem is an important personal resource in the behavioral protective factors of adolescents [28,29]. Meanwhile, some studies have shown that the feeling of hope has a protective effect on adolescents’ problem behaviors. Adolescents can respond positively to difficulties with the feeling of hope and decrease the likelihood of engaging in problem behaviors [30,31].

We found that the feeling of hope and self-esteem share a common element, namely confidence in the future. Confidence in the future is one’s expectation and hopes for a better life situation [32]. As an aspect of self-confidence, confidence in the future is not only a part of self-esteem [33] but also a part of the feeling of hope (called “dynamic thought” in Snyder’s hope theory) [34]. High confidence helps children cope with negative outcomes and worry less about failure [35]. However, it is not clear whether adolescents’ confidence in the future plays a role in adolescents’ problem behaviors and whether adolescents’ confidence in the future mediates the relationship between family socioeconomic status and adolescents’ problem behaviors.

### 1.4. The Current Research

This study was guided by the risk-protection model of the CTMPB and based on the premise Jessor proposed that family socioeconomic status was an important influencing factor of adolescent problem behaviors, further exploring the influencing mechanism of family socioeconomic status on adolescent problem behaviors. It examined the influence of family socioeconomic status on adolescent problem behaviors, and further examined the mediating effects of parental educational expectations and adolescents’ confidence in the future on the link between family socioeconomic status and adolescents’ problem behavior, namely, it examined whether parents’ educational expectations and adolescent problem behaviors are protective factors or risk factors of adolescents’ problem behaviors. Therefore, the hypotheses are proposed as follows:

**Hypothesis** **1** **(H1).** *Family socioeconomic status significantly negatively predicts adolescents’ problem behaviors.*

**Hypothesis** **2** **(H2).** *Parental educational expectations and adolescents’ confidence in the future play a parallel mediating role between family socioeconomic status and adolescents’ problem behaviors*.

**Hypothesis** **3** **(H3).** *Parental educational expectations and adolescents’ confidence in the future play a chain mediating role between family socioeconomic status and adolescents’ problem behaviors*.

## 2. Materials and Methods

### 2.1. Participants

This study used data from the 2014–2015 academic year of the China Education Panel Survey (CEPS) conducted by the China survey and data center at Renmin University of China. The survey uses a multistage probability-to-scale (PPS) sampling method. At the first sampling stage, 28 counties (districts) were selected from the national county (district) level administrative units. At the second sampling stage, four schools with grade 7 and/or grade 9 were selected from the geographical area of each enrolled county (district). At the third sampling stage, four classes were selected from each school, including two grade 7 classes and two grade 9 classes. A total of 438 classes in 112 schools were randomly selected from the selected county-level units. At the fourth sampling stage, all students, parents, head teachers, teachers of the main subject (English, Chinese), and school leaders of the enrolled class constitute the final survey samples. The study was approved by the Ethics Committee of Renmin University of China, and all participants were informed that their participation would be voluntary and that they were free to withdraw from the study at any time. The anonymity of the study was also emphasized before the data were collected. Participants were asked to respond independently according to their actual situation. Due to the missing data, a total of 6888 students in the second grade of junior high school were finally included in this study. Among them, 3342 (48.5%) were girls and 3546 (51.5%) were boys; their average age was 14.50 years (SD = 0.68 years).

### 2.2. Measures

#### 2.2.1. Family Socioeconomic Status

Family socioeconomic status was assessed based on the method developed by Bradley and Corwyn [13], which includes parents’ educational level, parents’ occupation, and family economic income as measurement indicators. Specifically, parents’ educational level was assigned based on the Programmer for International Student Assessment criteria [36], and scores ranging from 1, “no education” to 7, “graduate student or above” were given, with higher scores indicating higher levels of education. According to the method developed by Lu [37], the occupation of parents was classified into 10 occupational classes in contemporary Chinese society, with scores ranging from 1, “unemployed and laid-off” to 10, “leaders of government departments”. Family income was divided into five levels and scored 1–5, with higher scores indicating higher income. Finally, based on relevant studies [38,39], parents’ educational level and occupation scores were converted into standard scores. Family income scores and standard scores were the measurement indicators of family socioeconomic status.

#### 2.2.2. Problem Behaviors

Problem behaviors were measured by the problem behaviors questionnaire from the 2015 student questionnaire of the CEPS program. The questionnaire consisted of 10 questions, such as “Have you done the following behaviors in the past year? To fight” represented on a 5-point Likert scale, where 1 = “never”; 2 = “occasionally”; 3 = “sometimes”; 4 = “often”; 5 = “always”. The Cronbach’s coefficient of the whole scale is 0.83.

#### 2.2.3. Parental Educational Expectations

Parental educational expectations were measured with the question, “Which level of education do your parents want you to achieve in the future?” and corresponding answers from “give up school now” to “doctor”. After referring to another study on educational expectations [40], this study regarded the “indifferent” option corresponding to the “give up school now” option in the questionnaire as the lowest expectation. Technical secondary schools, technical schools, vocational high schools, and ordinary high schools are classified as belonging to the same grade because they have the same number of education years. Finally, there was a 7-point Likert scale, wherein higher scores indicate a higher level of education.

#### 2.2.4. Confidence in the Future

Confidence in the future was reflected by one item. Students were asked, “Do you have confidence in your future?” A 4-point Likert scale was used: 1 = “no confidence at all”, 2 = “less confident” 3 = “comparative confidence”, and 4 = “very confident”. The higher the score, the more confident the student is.

### 2.3. Data Analysis

This study used SPSS 26.0 and PROCESS V3.5. Model 6 in PROCESS V3.5 was selected for testing. Our analyses are presented in three parts, namely, descriptive statistics and correlations including means, standard deviations, and Pearson correlation coefficients of main variables; the regression models analysis including four equations and regression coefficients, standard errors, and *p* values, and mediating effect analysis including the ratio of three indirect effect sizes and their comparisons.

#### 2.3.1. Descriptive Statistics and Correlations

Table 1 presents the descriptive statistics and correlation analysis results of each variable. It shows that there were significant correlations between family socioeconomic status, parental educational expectations, adolescents’ confidence in the future, adolescents’ problem behaviors, and parental relationships. Problem behaviors were negatively correlated with family socioeconomic status, parental educational expectations, adolescents’ confidence in the future, and parental relationships. Gender was significantly correlated with parental educational expectations, adolescents’ problem behaviors, and parental relationships, but not family socioeconomic status, and adolescents’ confidence in the future.

Previous studies have found that gender and parental relationship have an impact on adolescents’ problem behaviors [41,42,43], so gender and parental relationship were controlled in this study as covariates and were included in the correlation analysis.

#### 2.3.2. Regression Models Analysis

The results of the correlation analysis meet the statistical requirements for further testing the mediating effect of parental educational expectations and adolescents’ confidence in the future [44]. Next, the SPSS macro program developed by Hayes [45] was used to perform the bootstrap-based mediating effect test, using model 6.

Table 2 shows the analysis results of the regression relationship among the variables, and equation 1 indicated that family socioeconomic status significantly negatively predicted adolescents’ problem behaviors (*b* = −0.125, *p* < 0.001). When parental educational expectations and adolescents’ confidence in the future were included in the regression equation, equation 2 indicated that family socioeconomic status positively predicted parental educational expectations (*b* = 0.191, *p* < 0.001); equation 3 indicated that family socioeconomic status positively predicted adolescents’ confidence in the future (*b* = 0.139, *p* < 0.001), parental educational expectations significantly positively predicted adolescents’ confidence in the future (*b* = 0.131, *p* < 0.001); equation 4 indicated that parental educational expectation significantly negatively predicted adolescents’ problem behaviors (*b* = −0.124, *p* < 0.001), adolescents’ confidence in the future significantly negative predicted problem behaviors (*b* = −0.162, *p* < 0.001), when family socioeconomic status was significantly negative in predicting adolescents’ problem behaviors (*b* = −0.074, *p* < 0.001).

#### 2.3.3. Mediating Effect Analysis

**Figure 1:**
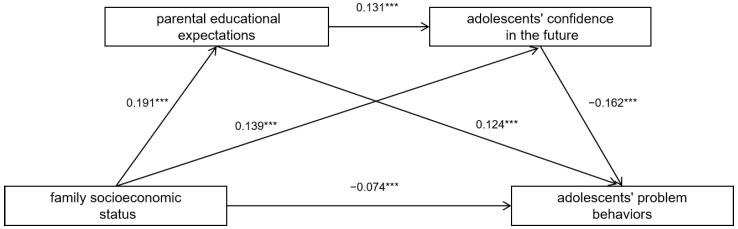
The path of multiple mediating effects. *** *p* < 0.001.

The results regarding the sizes of mediating effects (Table 3, Figure 1) show that parental educational expectations and adolescents’ confidence in the future played a significant mediating role in the link between family socioeconomic status and adolescents’ problem behaviors. The total standardized mediating effect size was −0.050. The mediating effect was composed of the following three indirect effects: Indirect effect 1: The path of family socioeconomic status, parents’ educational expectations, and adolescents’ problem behaviors (effect size, −0.024); indirect effect 2: The path of family socioeconomic status, adolescents’ confidence in the future, and adolescents’ problem behaviors 2 (effect size, −0.023); and indirect effect 3: The path of family socioeconomic status, parental educational expectations, adolescents’ confidence in the future, and adolescents’ problem behaviors (effect size, −0.004). The ratio of the three indirect effects to the total effect was 47.11%, 44.91%, and 7.98%, respectively. All three indirect effects were significant.

The indirect effect comparison option in model 6 was selected, and the indirect effects of different pathways were compared pairwise to investigate whether there were significant path differences in the pathways. Comparison 1 shows that the bootstrap 95% confidence interval of the difference between indirect effects 1 and 2 contained a 0 value, indicating that there was no significant difference between indirect effects 1 and 2. Using the same idea, the bootstrap 95% confidence intervals for the differences between indirect effects 1 and 3, and indirect effects 2 and 3 did not include a value of 0, indicating significant differences.

## 3. Discussion

This study used a nationally representative sample from China. It aimed at exploring the influence mechanism of family socioeconomic status on adolescents’ problem behaviors, guided by the CTMPB. We examined the influence of family socioeconomic status on adolescents’ problem behaviors and analyzed the mediating roles of parental educational expectations with adolescents’ confidence in the future.

### 3.1. The Influence Mechanism of Family Socioeconomic Status on Problem Behaviors

The findings of this study revealed that family socioeconomic status can significantly predict adolescents’ problem behaviors. Both parental educational expectations and adolescents’ confidence in the future play a parallel and chain mediating role between family socioeconomic status and adolescents’ problem behaviors.

First, the results of this study supported H1. Table 2 (“the equation 1”) indicated that the effect size (*b* = −0.125) and significance level (*p* < 0.001) of family socioeconomic status influence adolescents’ problem behaviors, which supported H1, namely, family socioeconomic status negatively predicts adolescents’ problem behaviors. This result is consistent with previous research results [15,20] and supports not only the CTMPB but also the bioecological theory. The bioecological theory suggests that although the exosystem (family socioeconomic status, in this study) cannot directly affect individuals, it can affect individuals through the microsystem (parental educational expectations, in this study) [46]. The results of this study showed the consistency of the CTMPB and the bioecological theory.

Second, the results of this study also supported H2. Table 3’s “indirect effect 1” (family socioeconomic status, parental educational expectations, and adolescents’ problem behaviors) indicated the effect size (*b* = −0.024) and significance level (Boot LLCI = −0.030, Boot ULCI = −0.017) of the path, and “indirect effect 2” (family socioeconomic status, adolescents’ confidence in the future, and adolescents’ problem behaviors) also indicated the effect size (*b* = −0.023) and significance level (Boot LLCI = −0.028, Boot ULCI = −0.017) of the path, which supported H2: parental educational expectations and adolescents’ confidence in the future play a parallel mediating role between family socioeconomic status and adolescents’ problem behaviors.

Equation 2 indicated that the effect size (*b* = 0.191) and significance level (*p* < 0.001) of family socioeconomic status influence parental educational expectations, which supported the family investment theory, which states that family socioeconomic status can positively predict parental educational expectations. Equation 3 indicated the effect size (*b* = −0.124) and significance level (*p* < 0.001) of parental educational expectations on adolescents’ problem behaviors, and thus, parental educational expectations can negatively predict adolescents’ problem behaviors. This suggests that high educational expectations of parents are a protective factor against adolescents’ problem behaviors.

Table 3’s “indirect effect 2” (family socioeconomic status, adolescents’ confidence in the future, and adolescents’ problem behaviors) confirmed that adolescents’ confidence in the future mediated the relationship between family socioeconomic status and adolescents’ problem behaviors. This result supports the measurement of risk factors and protective factors in the CTMPB. The CTMPB takes “low self-esteem” and “low perception of success in life” as the dimensions of the measurement scale to indicate the characteristics of the individual [47]. In addition, this result also supports the Hope Theory. Confidence in the future is a feeling of hope, which is a psychological protective factor [48] and has a protective effect on negative psychology [49,50].

Third, the results of this study also supported H3. Apart from the two separate mediating effect paths above, there is a third path. Table 3’s “indirect effect 3” (family socioeconomic status, parental educational expectations, adolescents’ confidence in the future, and adolescents’ problem behaviors) indicated the effect size (*b*= −0.004) and significance level (Boot LLCI = −0.005, Boot ULCI = −0.003) of the path, which supported H3, namely parental educational expectations and adolescents’ confidence in the future play a chain mediating role between the link of family socioeconomic status and adolescents’ problem behaviors. The effect size of this path is small but significant, which shows that parental educational expectations can also positively predict adolescents’ confidence in the future.

### 3.2. Implications for Adolescents’ Problem Behaviors Prevention and Intervention

The results of this study showed that high parental educational expectations are a protective factor against problem behaviors. The role of parental educational expectations may be more important than we thought. High parental educational expectations play the role of encouraging and monitoring adolescents’ growth. It can not only enhance adolescents’ confidence in the future but also decrease the likelihood of engaging in problem behaviors. Parents should pay more attention to their educational expectations of their children, especially parents of lower socioeconomic status families.

Meanwhile, the results of this study also suggest that adolescents’ confidence in the future is important for an adolescent’s development. Families and schools need to pay attention to adolescents’ confidence and improve adolescents’ confidence in the future, particularly for adolescents in lower socioeconomic status families. Since confidence in the future is a part of hope, according to Snyder’s Hope Theory, confidence in the future (namely, dynamic thought) can be enhanced by strengthening “path thought.” For example, helping adolescents establish future goals and finding clear ways to achieve their goals can enhance their confidence in the future [34,51]. Even their feeling of hope is more conducive to the adolescents’ development.

### 3.3. Limitations and Future Research Directions

This study has some shortcomings. The CTMPB mainly conducts static analyses, this study is only based on cross-sectional data. It is thus difficult to determine the causal relationship between variables. Future research could be enriched by dynamic analyses. Secondly, according to the CTMPB, there are three types of protection and three types of risk. Future research could be extended to study various types of protection and take both the protection and risk factors into account. We can further explore how parental educational expectations affect adolescents’ problem behaviors and find more measures to prevent and intervene in adolescents’ problem behaviors.

## 4. Conclusions

There is a significant association between family socioeconomic status and adolescents’ problem behaviors, and family socioeconomic status is an important factor affecting adolescents’ problem behaviors. Family socioeconomic status can predict adolescents’ problem behaviors, and adolescents with lower family socioeconomic status are more likely to engage in problem behaviors.

Parental educational expectations and an adolescent’s confidence in the future played a parallel mediating role in the link between family socioeconomic status and problem behaviors. Both parental educational expectations and adolescents’ confidence in the future are protective factors against adolescents’ problem behaviors, and increasing parental educational expectations and an adolescent’s confidence in the future can decrease the likelihood of engaging in problem behaviors. Meanwhile, parental educational expectations and an adolescent’s confidence in the future played a chain mediating role in the link between family socioeconomic status and problem behaviors. There is a significant association between parental educational expectations and an adolescent’s confidence in the future, and increasing parental educational expectations can enhance adolescents’ confidence in the future.

## Figures and Tables

**Table 1:** Means, standard deviations, and Pearson correlation coefficients of main variables.

	M	SD	1	2	3	4	5
1 Family socioeconomic status	0.28	3.69					
2 Parental educational expectation	6.53	1.92	0.193 ***				
3 Adolescents’ confidence in the future	3.15	0.70	0.166 ***	0.160 ***			
4 Adolescents’ problem behaviors	15.34	4.68	−0.129 ***	−0.180 ***	−0.201 ***		
5 Parental relationships	0.91	0.29	0.025 *	0.053 ***	0.108 ***	−0.113 ***	
6 Gender			−0.012	−0.081 ***	0.019	0.148 ***	0.030 *

Note: N = 6888; * *p* < 0. 05, *** *p* < 0.001.

**Table 2:** Regression equations.

Variables	Equation 1		Equation 2		Equation 3		Equation 4	
	*b*	*t*	*b*	*t*	*b*	*t*	*b*	*t*
Family socioeconomic status	−0.125 ***	−10.604	0.191 ***	16.224	0.139 ***	11.647	−0.074 ***	−6.304
Parental educational expectations					0.131 ***	10.881	−0.124 ***	−10.437
Adolescents’ confidence in the future							−0.162 ***	−13.737
Gender	0.150 ***	12.772	−0.081 ***	−6.840	0.028 *	2.394	0.143 ***	12.416
Parental relationships			0.050 ***	4.255	0.096 ***	8.200	−0.092 ***	−7.944
*R*	0.226		0.215		0.234		0.310	
*R* ^2^	0.051		0.046		0.055		0.096	
*F*	123.978 ***		110.924 ***		100.062 ***		146.169 ***	

Note: Equation 1 = adolescents’ problem behaviors as the dependent variable; equation 2 = parental educational expectations as the dependent variable; equation 3 = adolescents’ confidence in the future as the dependent variable; equation 4 = adolescents’ problem behaviors as the dependent variable. All variables in the model have been standardized. * *p* < 0.05. *** *p* < 0.001.

**Table 3:** Mediating effect size comparison.

	Indirect Effect Size	Boot SE	Boot LLCI	Boot ULCI	Percentage
Total indirect effect	−0.050	0.004	−0.058	−0.042	100%
Indirect effect 1	−0.024	0.003	−0.030	−0.017	47.11%
Indirect effect 2	−0.023	0.003	−0.028	−0.017	44.91%
Indirect effect 3	−0.004	0.001	−0.005	−0.003	7.98%
Comparison 1	−0.001	0.005	−0.010	0.008	
Comparison 2	−0.020	0.003	−0.026	−0.013	
Comparison 3	−0.019	0.003	−0.024	−0.014	

Note: Boot SE and Boot LLCI and Boot ULCI refer to standard errors and lower and upper limits of 95% confidence intervals for indirect effects estimated by the bias-corrected percentile bootstrap method (5000 times) respectively. Indirect effect 1: family socioeconomic status, parental educational expectations, and adolescents’ problem behaviors; Indirect effect 2: family socioeconomic status, adolescents’ confidence in the future, and adolescents’ problem behaviors; Indirect effect 3: family socioeconomic status, parental educational expectations, adolescents’ confidence in the future, and adolescents’ problem behaviors.

## Data Availability

The data presented in this study are available on request from the corresponding author. The data are not publicly available due to privacy.
